# Pharmacogenomics of TNF inhibitors

**DOI:** 10.3389/fimmu.2025.1521794

**Published:** 2025-05-21

**Authors:** Zainab Jan, Farah El Assadi, Dinesh Velayutham, Borbala Mifsud, Puthen Veettil Jithesh

**Affiliations:** ^1^ College of Health and Life Sciences, Hamad Bin Khalifa University, Doha, Qatar; ^2^ William Harvey Research Institute, Queen Mary University of London, London, United Kingdom; ^3^ Pharmacology and Therapeutics, Institute of Systems, Molecular and Integrative Biology, University of Liverpool, Liverpool, United Kingdom

**Keywords:** pharmacogenomics, genetic variants, TNF inhibitors, human leukocyte antigen, drug efficacy and safety

## Abstract

Tumor necrosis factor alpha inhibitors (TNFi) are biologic drugs that target TNFα, a key pro-inflammatory cytokine, to suppress disease activity and alleviate symptoms of various autoimmune diseases, including inflammatory bowel disease. This review focuses on the five US FDA-approved TNFi including the monoclonal antibodies Infliximab, Adalimumab, Golimumab, Certolizumab pegol and the soluble TNFα receptor fusion protein Etanercept, with a brief mention of other available biosimilars to TNFi. The review aims to summarize the recent evidence on the pharmacokinetics, pharmacodynamics, and pharmacogenomics of TNFi with a particular focus on Human Leukocyte Antigen (HLA) variants in terms of their genetic contribution to the response to TNFi. HLA variants have been linked to heterogeneity in the efficacy and safety of TNFi among patients. Building on the summarized evidence, the last part of the review discusses the potential clinical utility of testing for pharmacogenetic variants that are linked to the response to TNFi prior to the drug prescription, and it also addresses the future directions to achieve personalized treatment for TNFi users.

## Graphical Abstract

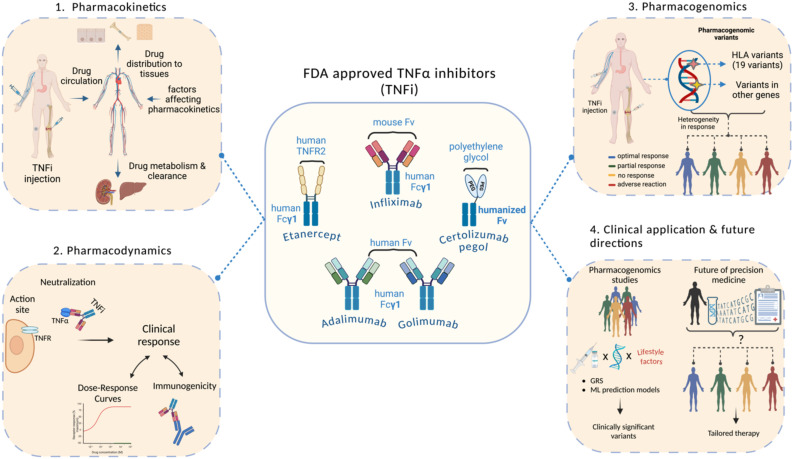

## 1 Introduction

**Figure 1:**
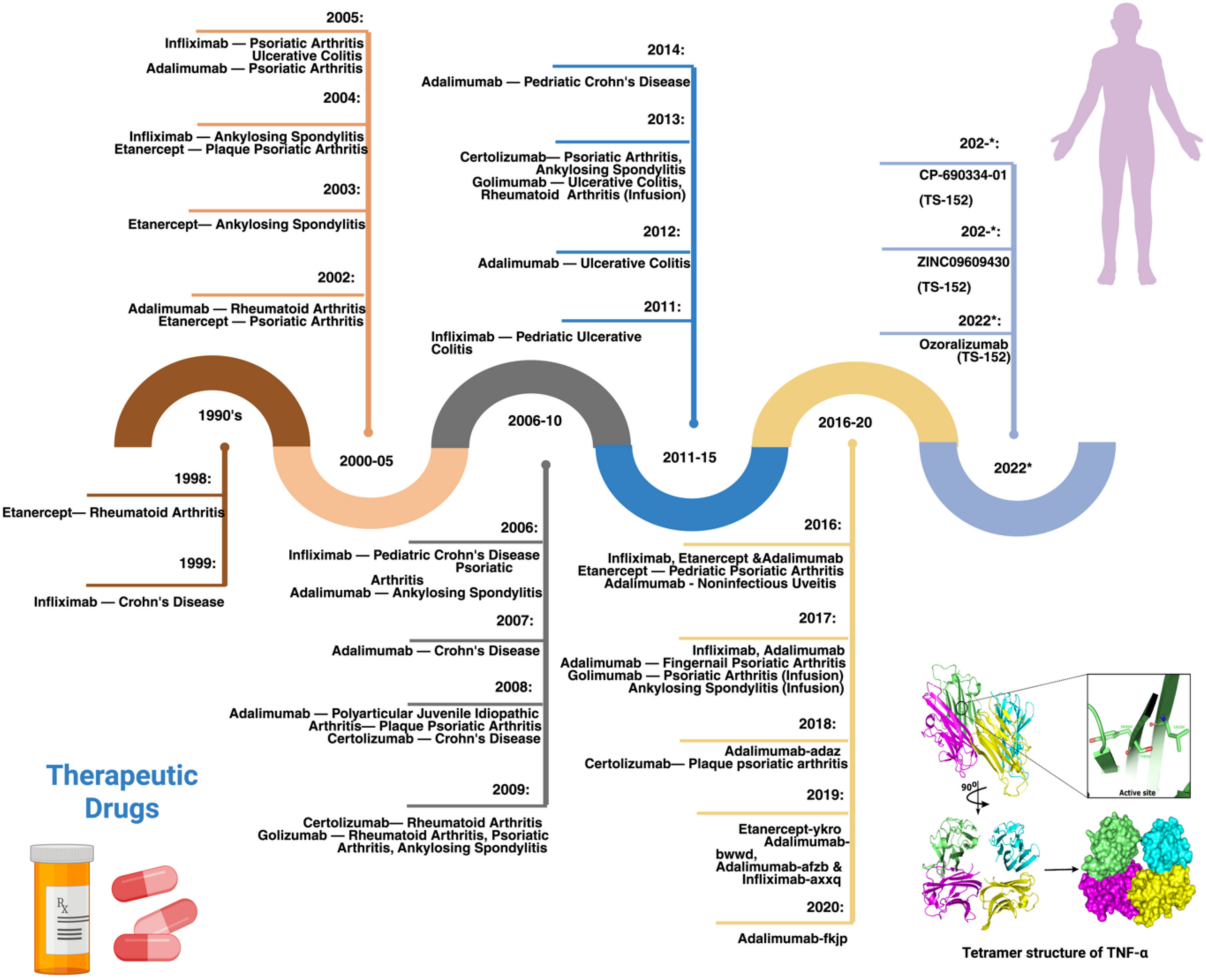
Timeline of the development and approval of TNF inhibitors along with the structure of TNFα. 1990s: In 1999, Infliximab was approved to treat Crohn’s disease (CD), later it was used for Rheumatoid Arthritis (RA), Ankylosing Spondylitis, and other autoimmune diseases. 2000-05: In 2002, Adalimumab was approved by the FDA to treat RA. Later, it was also used for CD, Psoriatic Arthritis, Juvenile Idiopathic and other diseases. 2006-10: In 2009, Golimumab was approved by the FDA to treat RA and used in combination with methotrexate, which was also later approved for Ankylosing Spondylitis, Ulcerative colitis, and Psoriatic arthritis treatment. 2011-15: In 2011, Infliximab was approved by the FDA for pediatric Ulcerative Colitis treatment, followed by Adalimumab and Golimumab; Adalimumab for CD. 2016-20: In 2018, Certolizumab pegol was used to treat RA, Psoriatic arthritis, CD, Ankylosing Spondylitis, and Psoriatic Arthritis. 2021-22: Ozoralizumab (TS-152) developed by Taisho research institute to treat RA.

Tumor Necrosis Factor alpha inhibitors (TNFi) are widely used in the clinical setting to treat severe autoimmune diseases as they have shown promising efficacy and safety (1). As the name suggests, TNFi are designed to block the action of Tumor Necrosis Factor alpha (TNFα), which is one of the most potent proinflammatory cytokines. TNFα has been associated with the pathogenesis of several autoimmune diseases (ADs) including rheumatoid arthritis (RA) (2–4), inflammatory bowel disease (IBD) (5–7), psoriasis (PS) (8, 9), and ankylosing spondylitis (AS) (10), serving as a driver of chronic inflammation (11). TNFα is a type II transmembrane protein produced mainly by macrophages and to a lesser extent by T and B lymphocytes, natural killer cells, and neutrophils (12, 13). It exists in a membrane-bound form (mTNFα) and a soluble form (sTNFα) (14). Both forms of TNFα bind to specific receptors known as TNFR1 (also known as TNFRSF1A or p55) and TNFR2 (also known as TNFFSRF1B or p75); however, sTNFα has less affinity to TNFR2 (15). The transmembrane form of TNF is the prime activating ligand of the 80 kDa tumor necrosis factor receptor (16). TNFR1 is universally present on the membranes of almost all nucleated cells while TNFR2 is primarily present on membranes of immune cells, endothelial cells, and some tumor cells (17, 18). Upon binding TNFR1 and TNFR2, TNFα initiates a complex cascade of molecular signals for various biological functions and different cellular responses including inflammation, cell death, cell proliferation, and differentiation (19, 20). The signaling cascade initiated upon the activation of TNFR1 by TNFα leads to the activation of the Nuclear factor kappa-light-chain-enhancer of activated B cells (NF-κB) (21) pathway and the mitogen-activated protein kinases (MAPK) pathway (22), which subsequently induce inflammation, immune response, tissue degeneration, and cell survival and proliferation. Furthermore, the binding of TNFα to TNFR1 can mediate cell death via apoptosis or necroptosis through activating caspase-8 or mixed lineage kinase domain-like protein (MLKL), respectively. In contrast to TNFR1, TNFR2 lacks a death domain and thus does not prompt cell death directly. However, similar to TNFR1, the activation of TNFR2 stimulates NF-κB, MAPKs, and protein kinase B (AKT) pathways, promoting cell proliferation, tissue regeneration, and inflammatory responses against pathogens (23). TNFα drives inflammation by stimulating the secretion of other inflammatory cytokines, activating immune cells, and amplifying its own expression through a positive feedback loop (24). The rationale behind TNFα being a suitable target in ADs lies in its central role in inflammation and that it is “at the apex” of the signaling cascade of the pro-inflammatory cytokines (25), along with its implication in the pathogenesis of several ADs. In addition, early experiments proving the efficacy of the first TNFi, infliximab, in AD patients (26), opened the door for developing and improving anti-TNF treatment as a therapeutic approach in several ADs gradually (**Figure 1**).

Adalimumab, Infliximab, Certolizumab pegol, Golimumab, and Etanercept are the five TNFi approved by the US Food and Drug Administration (FDA) (27). The timeline for the development of TNFi has been comprehensively discussed in previous reviews (20, 28), from which we adapted and modified the figure presented here (**Figure 1**). Apart from Etanercept, which is a fusion protein that serves as a receptor for TNFα, the other four TNFi are monoclonal antibodies (mAbs). Each TNFi has two functional regions: the constant region (Fc) and the variable region (Fab), except for Certolizumab pegol, which lacks the constant region. When the Fab region of the mAb binds to TNFα, it prevents TNFα from interacting with its receptors, which is the therapeutic objective of this family of compounds (13). Although these inhibitors have been used since the past decade, the complete mechanism of action is still unclear. Previous studies have reported that TNFi neutralizes TNFα, induces direct and indirect apoptosis, modulates the immune system, induces Fc-dependent apoptosis, and promotes outside-to-inside signaling. The inflammation is reduced by the immediate neutralization reaction driven by the TNFi, which inactivates the proinflammatory cytokine TNFα (29). Nonetheless, these inhibitors appear to have more complex activities than simple blocking, owing to the complexity of the TNFα signaling. TNFi have substantially improved the treatment course of autoimmune diseases. However, response to TNFi is significantly variable, with up to 40% of patients not experiencing a positive clinical outcome. This lack of response can be classified as primary, where patients fail to respond from the outset, or secondary, where the response diminishes over time despite initial effectiveness (30).

Pharmacogenomic (PGx) variants influence how individuals respond to drugs. Variable responses to medications among ethnic groups have been attributed to the diversity of PGx variants. Previous studies highlighted that approximately 20-30% of the variability in drug efficacy and toxicity can be better elucidated by exploring PGx variants associated with drug response (31). Genetic variants have been identified in pharmacogenes that affect both pharmacokinetics and pharmacodynamics of specific drugs. Moreover, previous studies have identified that more than 90% of patients carry at least one variant in pharmacogenes that prompts a change in dosage or drug selection (32, 33). After identifying genetic loci associated with drug responses, it becomes imperative to analyze the distribution of these variants in populations, particularly because some variants, such as the human leukocyte antigen (HLA) variants, increase the risk of severe adverse effects in response to some medications. This review discusses the pharmacokinetics, pharmacodynamics, and pharmacogenomics aspects of TNFα inhibitors, with a particular focus on HLA variants.

## 2 Pharmacokinetics of TNF inhibitors

**Figure 2:**
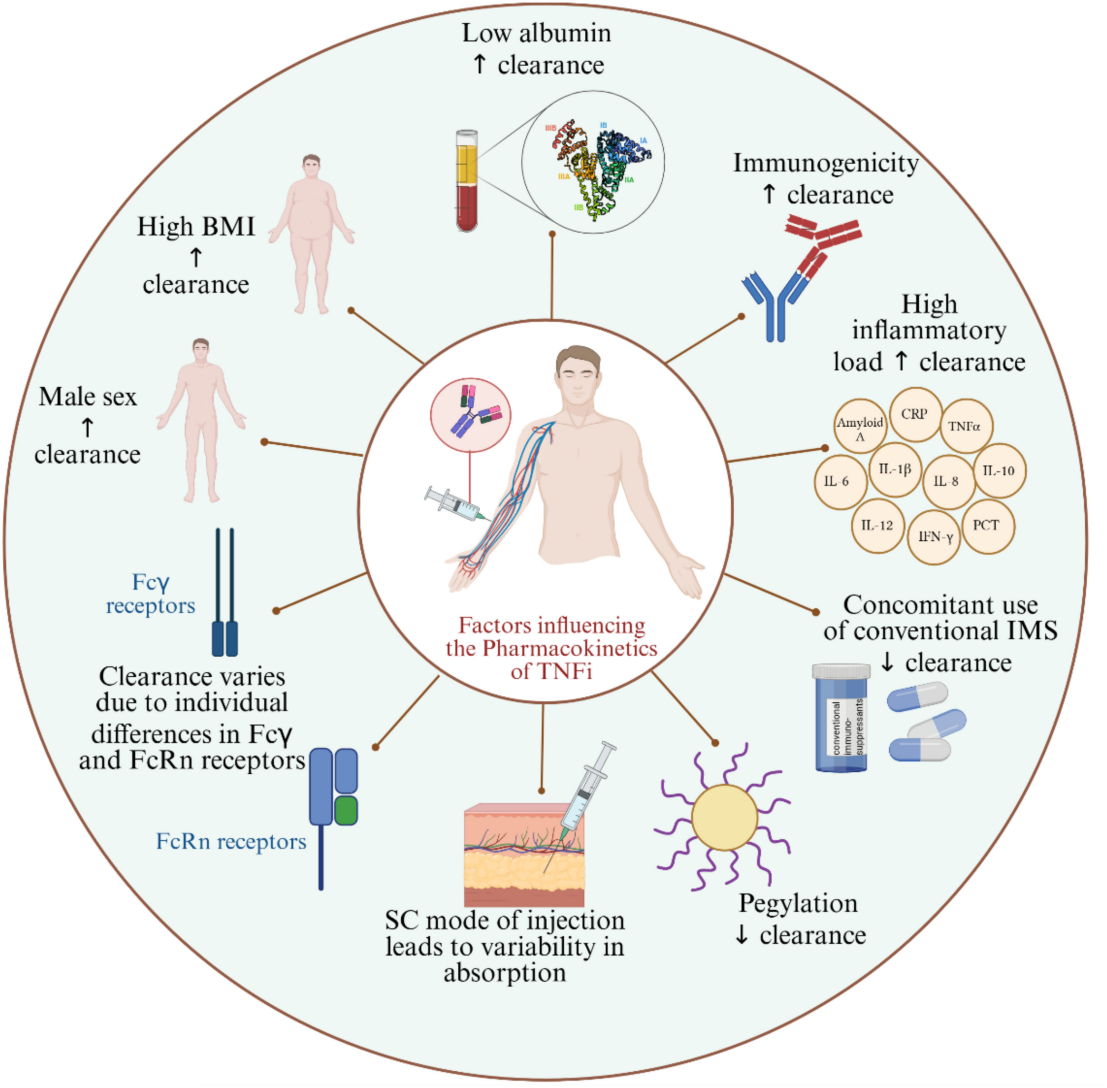
Factors influencing the pharmacokinetics of TNFi. BMI, Body Mass Index; SC, Subcutaneous; IMS, Immunosuppressants; FcRn, Neonatal Fc receptor; ↑: increases; ↓: decreases.

TNF inhibitors are protein compounds that target either membrane-bound or soluble TNF (34). These medications have complex pharmacokinetics and pharmacodynamics features due to the high affinity to their pharmacological target, compared to small-molecule medicines (35). Since TNFi are large protein molecules with low membrane permeability, they are poorly absorbed in the gastrointestinal tract. Therefore, they are parenterally administered and distributed via the lymphatic system before being metabolized and broken down into small molecules. Biologics that target soluble TNF, such as Etanercept, have a linear elimination profile, whereas biologics that target membrane-bound antigen have a non-linear elimination profile (36). The non-linear elimination of the latter category of biologics is related to target-mediated drug disposal (TMDD). One important pharmacokinetic property of TNF inhibitors is half-life (37). Etanercept has the lowest half-life of 3–5.5 days, while all other inhibitors have half-lives of ~ two weeks (38). The frequency of administration of TNFi is associated with its half-life, wherein a short half-life warrants more frequent administration. Infliximab is injected intravenously, while all other TNF inhibitors are used both intravenously and subcutaneously (39). The pharmacokinetics of TNFi is influenced by several variables as detailed in **Figure 2**.

The response to TNFi is influenced by pharmacokinetic variables. Males appear to have higher clearance of TNFi compared to females (40). In general, the sex-related pharmacokinetic differences are attributed to molecular and physiological differences between males and females. To our knowledge, the available evidence on the TNFi pharmacokinetic discrepancies between males and females is observational rather than investigating the mechanisms in effect (41, 42). One plausible factor for higher drug clearance in males is that females naturally have more body fat (43); the logic behind it is that the blood circulation in fat tissues is poor, thus having more fat composition contributes to variations in drug kinetics (44). Another possible reason is the difference in innate and adaptive immune responses between males and females; for instance, the peripheral blood mononuclear cells and neutrophils (45) in men were shown to produce more TNFα than in women after stimulation by lipopolysaccharide; hence, when TNFi is bound to its target, it is cleared faster. On the contrary to normal levels of body fat, excess body fat is correlated with lower serum levels of TNFi, whereby studies have shown that people with high body mass index (BMI) have lower trough levels of TNFi compared to individuals with normal weight (46). Evidence on the mechanism behind the inverse association of obesity and serum TNFi levels is scarce; however, some studies attributed it to the amplified inflammatory status induced by obesity and to a phenomenon known as TNF sink or antigen sink (47, 48). Briefly, in an inflammatory state, the anti-inflammatory drug, in this case, TNFi, can bind to all TNF in the blood, and the unbound TNFi will be sequestered by the saturated TNF via a pattern described as a sink; studies found that this phenomenon leads to rapid clearance of TNFi (48). Furthermore, low albumin levels (49) and high levels of inflammatory biomarkers, such as C-reactive protein (CRP) (50), are associated with higher clearance of TNFi. To our knowledge, the link between hypoalbuminemia and the reduced clearance of TNFi is not clearly elaborated. It is well-established that albumin is the most abundant serum protein and that low albumin levels are associated with severe inflammatory status. One of its key roles is binding drugs and other molecules in the blood; although it does not bind large molecules, such as TNFi, it was shown to have a protective effect against the degradation of drugs, including TNFi (49, 51). This could explain why low albumin level is linked to higher clearance of TNFi. On the other hand, high CRP levels reflect the presence of inflammation, and its production is usually triggered by enhanced TNFα production as is the case in systemic inflammatory diseases; therefore, a high CRP level is linked to high serum TNFα levels, which consequently lead to accelerated clearance of TNFi (50). Another well-established factor implicated in TNFi kinetics is the development of anti-drug antibodies (ADAs), whereby lower trough levels of TNFi were reported in presence of ADAs (52). ADAs can neutralize TNFα by binding to the pharmacologically active site of the TNFi, or they can bind to TNFi without neutralizing it; both cases lead to enhanced clearance of TNFi (53–55). On the contrary, some studies found that the concomitant intake of immunomodulators (56) as well as the pegylation of TNFi (57) are linked to reduced clearance and higher drug levels. The use of concomitant immunosuppressives could prevent ADAs production and consequently lead to prolonged presence of TNFi in serum and slower clearance (58, 59). More studies are required for conclusive evidence on some of these variables, such as the concomitant use of immunomodulators (60). In terms of PEGylation, the hydrophilic nature of PEG molecules improves the solubility of therapeutic proteins, such as TNFi, and promotes higher accumulation at target sites through enhanced permeability (61, 62). In fact, PEGylation can also protect therapeutic proteins, such as TNFi, from proteolytic degradation, thereby reducing their clearance (63). Clearance of TNFi has also been shown to vary according to differences in the neonatal Fc receptor (FcRn) (64) and Fcγ receptors (65). For instance, two alleles out of five in a variable number of tandem repeats in FcRn, showed lower levels of TNFi upon initiating the treatment, whereby patients with VNTR2/VNTR3 genotype had lower levels of infliximab or adalimumab in their serum compared to patients with VNTR3/VNTR3 genotype (64). The Fcγ receptors might accelerate clearance through an increased binding of the Fc portions of therapeutic mAbs to high affinity FcgRs inducing their elimination and resulting in a rapid decrease in serum drug levels (66). Subcutaneous administration of TNFi was also found associated with variabilities in absorption and, consequently, the trough levels of the drug (67). The primary limitations of subcutaneous administration include the restriction on the volume that can be delivered (typically no more than 1 mL) compared to the intravenous route, and the heterogeneity in dosage absorption among patients, ranging from 50% to 100%, which result in greater pharmacokinetic variability between individuals and doses (60). The subcutaneous administration also slows down the absorption of the drug due to longer processing of foreign bodies in the skin (60). One study reported more stable drug levels upon subcutaneous infliximab administration compared to intravenous infliximab (68), while another study showed that intra-articular injection of TNFi was more effective than subcutaneous TNFi administration (69).

## 3 Pharmacodynamics of TNF inhibitors

Monoclonal TNFi antibodies interact with membrane bound TNFα, while the fusion proteins like Etanercept engage with free soluble TNF. These mechanisms prohibit TNFα from binding to its own receptors on the inflammatory cells. The interaction of TNFi with TNFα is known as TNFα neutralization, by which downstream inflammatory cytokines, such as IL-1 and IL-6, are subsequently suppressed, and the inflammatory processes subside (70). Although TNFi appear to have a selective and precise anti-inflammatory action, they can cause significant side effects such as infections and malignancies (70). This could be due to TNFα’s involvement in normal physiological processes, including tumoricidal activities and host defense. Because monoclonal antibodies can be directed against a wide range of soluble or membrane-bound targets, they can exert their pharmacological effects through several ways. Soluble TNFα exists as a circulating cytokine and is involved in systemic inflammation (71). mAbs such as infliximab, adalimumab, and golimumab primarily target soluble TNFα, neutralizing its pro-inflammatory effects by preventing its interaction with TNF receptors on immune cells. This leads to the inhibition of downstream signaling pathways, particularly the NF-κB and MAPK pathways, which are critical for the activation of immune responses, inflammation, and the recruitment of other immune cells to sites of infection or injury (72, 73). On the other hand, membrane-bound TNFα is expressed on the surface of certain immune cells, such as T-cells, macrophages, and dendritic cells (74). mAbs like etanercept, which acts as a soluble TNF receptor fusion protein, bind to both soluble and membrane bound TNFα (75). By blocking membrane-bound TNF, etanercept can modulate immune cell interactions differently than mAbs targeting only soluble TNFα. This may impact the activation of various immune responses, such as cell-mediated immunity, through the reverse signaling mechanism of membrane-bound TNF, which can influence the activation of both pro-inflammatory and anti-inflammatory pathways (29, 76). Targeting soluble TNFα can primarily dampen systemic inflammation, while blocking membrane-bound TNFα could alter immune cell functions more directly by affecting cell-cell communication and immune cell activation (77).

**Figure 3:**
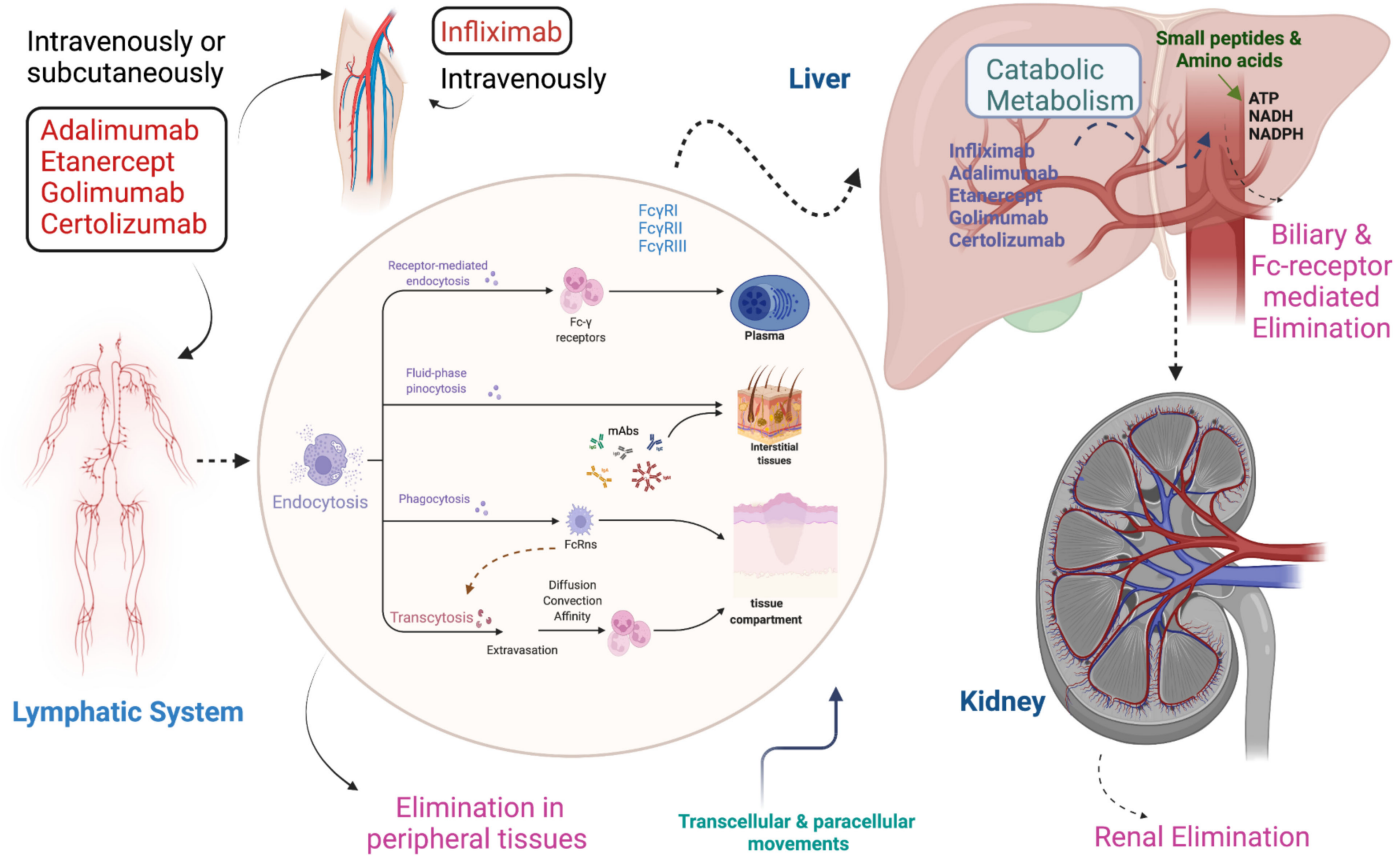
Pharmacokinetics and pharmacodynamics of TNF inhibitors. Infliximab is injected intravenously while other TNFi such as Adalimumab, Golimumab, Etanercept, and Certolizumab pegol are given intravenously and subcutaneously. All TNFi circulate via the lymphatic system and absorb into the body by endocytosis. Absorption occurs through different mechanisms, including receptor-mediated endocytosis, fluid phase pinocytosis, phagocytosis, and transcytosis. Metabolism occurs in the liver, while elimination takes place from the body via three different ways: 1) Peripheral tissues, 2) Biliary & Fc-receptor 3) Renal.

Moreover, the TNFi are administered parenterally and are absorbed into the bloodstream through mechanisms such as diffusion and facilitated transport at the injection site. Intravenous (IV) administration of TNFi offers 100% bioavailability since the drug is delivered directly into the bloodstream, leading to a quicker onset of action (78, 79) (**Figure 3**). In contrast, subcutaneous (SC) administration typically results in slower absorption and lower bioavailability, but it allows for more gradual drug delivery over time (80). Studies comparing the two routes, such as those involving infliximab (IV) and adalimumab (SC), have shown comparable therapeutic efficacy, although IV formulations are more commonly associated with infusion-related reactions (IRRs) including fever, chills, and shortness of breath (81). SC administration, on the other hand, is generally linked to local injection site reactions, such as pain or redness (82), but carries a lower risk of IRRs. Additionally, while both delivery methods activate the immune system similarly, the faster delivery associated with IV administration may lead to a more immediate immune response, whereas SC administration offers a more gradual immune modulation (83).

## 4 Safety and efficacy of TNF inhibitors

Some rare reports have been published that describe the adverse reactions of TNFi treatment, including tuberculosis (TB), heart failure, skin cancer, lymphoma, and the occurrence of demyelinating diseases (65). Some recent studies reported that treatment with TNFi does not increase the chance of cancer in RA patients. A study reported that all TNFi are effective; however, Etanercept is better in terms of safety compared to other TNF blockers as minimal side effects were reported (84). Another study unraveled the effectiveness and safety of anti-TNFα therapy for Juvenile Idiopathic Arthritis and reported that Adalimumab has good efficacy and safety compared to Infliximab. In contrast, Infliximab has higher efficacy as compared to Etanercept. However, Golimumab and Certolizumab have lower efficacy than these three drugs (1). A higher risk of serious infections was associated with Infliximab, Certolizumab, and Adalimumab, whereas Etanercept contributes to a lower risk of discontinuation due to adverse reactions (85). A study involving a cohort of over 42 spondyloarthritis (SpA) patients found that anti-TNF therapy demonstrated acceptable safety and a positive response rate in SpA patients (86). The safety profiles of TNFi vary among ankylosing spondylitis and rheumatoid arthritis patients. A meta-analysis conducted on ankylosing spondylitis patients found that Adalimumab and Infliximab have good clinical outcomes with Infliximab being more effective compared to Adalimumab (87). There is limited evidence on golimumab, but one study observed low infection rate during treatment with golimumab in a patient that had RA (88).

## 5 Heterogeneity in response to TNF inhibitors

About 30% of patients initially fail to respond to anti-TNFα therapy (primary non-responders) and up to 50% of patients lose their response during the course of treatment (secondary non-responders). The substantial proportions of patients experiencing failure of TNFi treatment whether upon initiation or at a later stage highlight the need for biomarkers that can predict response to these inhibitors. One possible clinical biomarker is serum calprotectin, which is also known as S100A8/A9 or MRP8/14 complex. A study conducted in three cohorts, one with Infliximab, one with Adalimumab, and one with Rituximab showed that TNFi responders had considerably greater baseline serum calprotectin levels than non-responders in rheumatoid arthritis patients. The study reported that the MRP8/14 (myeloid-related protein) levels were frequently higher in those who responded to targeted therapy, regardless of the mechanism of TNFi (89). Another plausible predictor could be the baseline TNFα levels; in a study conducted on 36 Crohn’s disease patients, it was found that primary non-response was related to higher TNF levels at baseline (56). Apart from clinical biomarkers, the links between genetic variants and response to TNFi were also assessed to identify genetic biomarkers. The genetic profiles of primary non-responders (PNR) have been investigated by previous studies by targeting the genes that are associated with cytokines and their receptors. For instance, associations were identified between non-response to TNFi and different genetic variants in *TNFRI*, *IL13R2*, *VNTR2*/*VNTR3*, *IL23R*, *MAPK* and *FcYRIIIa* (90). The secondary failure of response has been attributed in some cases to toxicity and immunogenicity. The risk of developing anti-drug antibodies is associated with several factors, including patient-related and treatment-related factors. The latter involves administration route, dosage, frequency, concomitant use of immunomodulators, and past TNFi treatment. While the individual-based factors that have been linked to immunogenicity include genetic susceptibility (91, 92), disease activity (93), BMI (56, 93, 94), and smoking status (56, 95),from a genetic perspective, the link between HLA markers in particular and immunogenicity is well-documented.

## 6 HLA variants and association with efficacy and toxicity to TNF inhibitor response

Adverse drug reactions (ADRs) are a major concern in healthcare. The presence of specific alleles in the human leukocyte antigen (HLA) system has been linked to the development of idiosyncratic ADRs. Understanding these connections is critical for prioritizing patient safety and customizing drug therapies. The HLA gene is the most extensively studied genetic biomarker for predicting therapeutic outcomes of rheumatoid arthritis (RA). The *HLA-DRB1* gene, a disease susceptibility marker, accounts for 30-50% of the genetic risks in RA. A distinct sequence at positions 70-74, termed shared epitope, is associated with the onset and pathology of RA (96). Shared epitope positivity was found to be linked to progressive joint destruction, particularly when valine is present at position 11 of HLA-DRB1. In terms of TNFi efficacy, a study using a UK RA cohort found significant disease activity improvement with TNFi therapy when valine, lysine and alanine were at positions 11, 71, and 74 of HLA-DRB1, respectively (97). Moreover, the HLA-DQA1*05 allele has been recognized as a risk factor for the development of antibodies against anti-TNF agents. HLA-DQA1*05:01 and its extended haplotypes were associated with infliximab immunogenicity, but not adalimumab whereas HLA-DQA1*05:05 and its extended haplotypes were associated with immunogenicity to both adalimumab and infliximab, with a stronger effect for adalimumab (98). Similarly, the *HLA-DRB9* g.32465390 G>T (rs2395185) G variant was also associated with IBD risk and primary non-response to Infliximab in a subset of patients with an age at diagnosis under 21 years (99). Moreover, other HLA variants such as HLA-DQA1 g.32622994 T>A/T>C (rs2097432) and HLA-DRB1 g.32465390 G>T (rs2395185) were associated with long-term anti-TNF drug response in children with IBD, highlighting their potential as predictive biomarkers for therapy outcomes (100). Previous studies recommend testing HLA-DRB1*3, HLA-DQA1*05:01, and HLA-DQA1*05:05 to predict immunogenicity to anti-TNF drugs; prior genetic testing for these variants could aid in therapy selection and preventive strategies (101). Moreover, another study reported that HLA-B rs41563412-GCA (p.Ser33LeufsTer9) homozygous carriers did not respond to adalimumab whereas HLA-DRB1 rs1071752-C (p.Gln125Gln) heterozygous and homozygous carriers showed response to infliximab (102). These two variants are situated in the exonic and intronic regions of the *HLA-DRB1* and *HLA-B* genes, respectively; both are an integral part of the adaptive immune system. These genes encode pivotal proteins for presenting antigens to CD8+ (in MHC-1) and CD4+ T-cells (MHC-II) in a dependent manner, shaping the altered adaptive immune response observed in Crohn’s disease (CD) patients. While genetic factors influencing clinical CD phenotypes may diverge from those related to anti-TNF response, the HLA region stands as a prominent risk locus for CD. Numerous genetic association studies have underscored the significant association between variants in *HLA-B* and *HLA-DRB1* with the development of CD. In contrast to CD4+ T-cells, antigen presentation to CD8+ T-cells follows MHC-I restrictions. Specifically, the HLA-B g. 31356966 (rs41563412) variant, located in the intronic region of the *HLA-B* gene, aligns with this MHC-I family. Notably, the sole homozygous carrier of the pathogenic *HLA-B* rs41563412-GCA variant, featuring a frameshift mutation (p. Ser33LeufsTer9), exhibited unresponsiveness to anti-TNF therapy. This suggests the potential involvement of this loss-of-function variant in modulating the CD8+ cytotoxic immune response, emphasizing its role in therapeutic outcomes (102).

Pharmacogenomics studies principally focusing on discovering variants in the HLA region and their associations with TNFi response, are reviewed in **Table 1** (98, 100, 103–110, 115). The assessment of genetic profiles of autoimmune patients is essential for identifying such HLA variants to enhance clinical diagnosis and implement personalized treatment.

**Table 1:** HLA variants associated with efficacy and toxicity of TNF inhibitors.

Drugs	Gene	SNP ID/Haplotype	Disease	Ethnicity	Clinical findings	References
Infliximab and Adalimumab	*HLA-DQA1*	HLA-DQA1*05	Crohn’s Disease (CA)	Europeans	Carriage of HLA-DQA1*05 almost doubles the rate of anti-TNF antibody development, independent of immunomodulator use for both infliximab and adalimumab	(98)
Infliximab	*HLA-B*, *HLA-C*, *HLA-DPB1*, *HLA-DQB1*, *HLA-DRB1*	HLA-B*39:01, HLA-B*08:01, HLA-C*12:03, HLA-DPB1*10:01, HLA-DQB1*02:01, HLA-DRB1*03:01, HLA-DRB1*04:04	Autoimmune disease	Europeans	These HLA variants are associated with infliximab response and susceptibility to drug-induced liver injury (DILI)	(103)
Adalimumab, Certolizumab pegol, Etanercept, Infliximab	*HLA-E*	HLA-E g. 30490287 G>A (rs1264457)	Rheumatoid Arthritis (RA)	Europeans	Genotype AA is associated with better responses to TNF inhibitors as compared to AG and GG	(104)
TNF inhibitors	*MICA*	MICA g. 31411200 (rs1051792)	Rheumatoid Arthritis (RA)	Europeans	The MICA rs1051792 GG genotype is associated with reduced response to TNF-blockade therapy in rheumatoid arthritis patients while the heterozygous GA genotype shows better therapeutic outcomes.	(105)
Infliximab	*HLA-DQA1*	HLA-DQA1*05 (rs2097432)	Crohn’s disease (CD)	Japanese	HLA-DQA1*05 (rs2097432) is a significant predictor of infliximab persistence (continued use or effectiveness of infliximab therapy over time), indicating sustained therapeutic response.	(106)
Adalimumab	*HLA-DRB1*	HLA-DRB1*03	Rheumatoid Arthritis (RA), Hidradenitis Suppurativa (HS), Inflammatory Bowel Disease (IBD)	Americans and Europeans	HLA-DRB1*03 allele is a significant predictor of anti-adalimumab antibodies formation in RA, HS, and IBD patients treated with anti-TNF therapies, contributing to variability in immunogenic responses	(107)
Infliximab and adalimumab	*HLA-DQA1*	HLA-DQA1*05	Inflammatory Bowel Disease (IBD)	European	HLA-DQA1*05 carrier status did not impact clinical or biochemical remission or treatment persistence in patients undergoing proactive drug monitoring with TNF inhibitors	(108)
TNF inhibitors	*HLA-DRB1*	HLA-DRB1*04:04	Rheumatoid Arthritis (RA)	European	Patients with HLA-DRB1*04:04 showed reduction in disease activity score after being treated with TNFi	(109)
Infliximab	*HLA-DQA1*	HLA-DQA1*05	Inflammatory bowel disease (IBD)	Canadian	HLA-DQA1*05 is linked to a higher risk of developing infliximab antibodies, leading to treatment failure and discontinuation in IBD patients	(110)
TNF inhibitors	*HLA-B*	HLA-B27	axial spondylarthritis (axSpA)	Europeans	HLA-B27 positivity is a predictor of better TNF inhibitor response in axial spondylarthritis	(111, 112)
TNF inhibitors	*HLA-C*	HLA-C*06:02	Psoriasis	European	HLA-C*06:02 is associated with poor response to TNFi	(113)
Adalimumab	*HLA-DQA1*	HLA-DQA1*05:05	Inflammatory Bowel Disease (IBD)	Europeans and non-Europeans	HLA-DQA1*05:05 is associated with increased risk of immunogenicity and low drug serum concentrations in TNFi-treated IBD patients	(114)

## 7 Other pharmacogenomic variants associated with TNF inhibitors

Several clinical and genome-wide association studies investigated the link between genetic variants and response to TNFi across various autoimmune diseases (**Table 2**). A meta-analysis, focused on spondyloarthropathy, psoriasis and Crohn’s disease, identified that the G allele in *TNF* -238G>A (rs361525), *TNF* -308G>A (rs1800629) and the C allele in *TNF* -857C>T(rs1799724) showed better response to TNFα blockers in the European population, but failed to show a significant impact in the Asian population (119, 144). Another meta-analysis conducted on individuals of European population assessed the association between SNPs and response to anti-TNF-α therapy across major autoimmune diseases, including psoriasis, rheumatoid arthritis, inflammatory bowel disease, and spondyloarthritis. They identified six SNPs in the *FCGR2A* (rs1801274), *FCGR3A* (rs396991), *TNF* (rs361525, rs1800629, rs1799724), and *TNFRSF1B* (rs1061622) genes that were significantly associated with treatment response, primarily within disease-specific subgroups. However, no single pharmacogenetic marker was consistently predictive across all conditions, highlighting the need for further research to identify robust biomarkers for guiding anti-TNF therapy (120).

**Table 2:** Genetic variants outside the HLA region associated with TNF inhibitor response.

Drugs	Gene	SNPs ID	Disease	Ethnicity	Clinical findings	References
Infliximab	*FTO*	FTO g.54026293G>A (rs7195994)	Rheumatoid arthritis (RA)	European	Variants at *FTO* gene are associated with infliximab response in RA patients	(116)
Infliximab	*TLR2, IL6, TNFRSF1B*	TLR2 c.597T>C (rs3804099), TLR2 g.153688371T>C (rs1816702), IL6 6331T>C (rs10499563), TNFRSF1B g.12207208 A>G (rs1061624)	Crohn’s disease (CD)	European	rs1816702, rs3804099, and rs1061624 are associated with a long-term response to infliximab, whereas rs10499563 C is linked to supratherapeutic infliximab levels	(117)
Infliximab, Adalimumab, Etanercept, Golimumab	*ADAM17*	*ADAM17* g.9504593 C>T (rs117645314), *ADAM17 g.*9550677T>A (rs117179141)	Rheumatoid arthritis (RA)	Korean	significant association with TNF inhibitors	(118)
Infliximab, Adalimumab	*TNF*	TNF g.31575254 G>A (rs1800629), TNF g.31575324 G>A (rs361525), TNF g.31574705 C>T (rs1799724)	Spondyloarthropathy, Psoriasis, Crohn’s disease (CD)	European	The C allele in rs1799724 & the G allele in rs1800629 and in rs361525 showed a better response	(119)
TNF inhibitors*	*TNF*	TNF g.31575254 G>A(rs1800629)	Psoriasis	European	G allele of rs1800629 is associated with good response as compared to A	(120)
TNF inhibitors*	*TNF* *TNFRSF1B*	*TNF* -857C TNFRSF1B 676T	Psoriasis	European	Individuals who carry alleles C at -857 on TNF and T at 676 on TNFRSF1B are significantly associated with positive response to etanercept	(121)
Adalimumab, Infliximab, Etanercept	*IL1B, LY96, TLR2, TLR9*	*IL1B* g. 112838252 C>G (rs1143623), *IL1B* g. 112836810 G>A (rs1143627), *LY96* g. 73989727 C>G (rs11465996), *TLR2* g. 153700794 C>A (rs11938228), *TLR2* g. 153685974 T>A (rs4696480) and *TLR9* g. 52224356 T>C (rs352139)	Psoriasis	European	These variants were associated with response to Adalimumab, Infliximab and Etanercept	(122)
TNF inhibitors*	*TLR4, IL1B, IL6, TNFRSF1A, TLR4, TLR2, LY96, IFNG, TLR9, MAP3K14*	*TLR4* g. 117712004 G>A (rs5030728), *IL1B* g. –3737G>A (rs4848306), IL6 g.- 6331T>C (rs10499563), *TNFRSF1A* g.- 609G>T (rs4149570), *TLR4* G>A (rs5030728), *TLR2* 597T>C (rs3804099), *TLR2* C>T (rs1816702), *LY96–*1625 C>G (rs11465996), *IFNG* 874T>A (rs2430561), *TLR9* -1486T>C (rs187084), *MAP3K14* T>C (rs7222094)	Inflammatory bowel disease (IBD)	European	These variants were associated with response to TNF inhibitors in IBD patients	(123)
TNF inhibitors*	*NKG2D*	NKG2D g.10379727A>G(rs2255336), NKG2D g.10372766C>G (rs1049174)	Rheumatoid arthritis (RA)	European	NKG2D rs1049174 and rs2255336 heterozygous genotypes are associated with better response to anti-TNF therapy in RA, while homozygous genotypes correlate with poor response	(124)
Adalimumab	*ATG16L1*	*ATG16L1 g.*233250193 (rs10210302)	Crohn’s Disease (CD)	European	TT & CT genotypes of rs10210302 are associated with good response as compared to CC	(125)4/25/25 3:09:00 PM
TNF inhibitors*	*TNF*	TNF -1031TT, TNF -238GA/AA, -857 CT/TT	Psoriasis	European	TNF -1031TT genotype and TNF -238GA/AA, -857CT/TT genotypes are associated with better response to anti-TNFi	(113)
TNF inhibitors*	*TNF*, *TNFRSF1A*	TNF -1031C/-308G, TNFRSF1A c.625 + 10A>G	Ankylosing Spondylitis	European	TNF-1031C/-308G haplotype is protective, while TNFRSF1A c.625 + 10A>G may predict delayed response to TNF-inhibitors in SpA patients	(126)
Infliximab, Adalimumab, Etanercept	*TNFRSF1A*	*TNFRSF1A g.*6341779 T>C (rs767455)	Rheumatoid and psoriatic arthritis	European	Genotype TT increased response, whereas CT and CC showed a decrease in response	(127)
Infliximab, Adalimumab, Etanercept	*PTPRC*	*PTPRC g.*198731313 G>A (rs10919563), FCGR2A R131H	Rheumatoid arthritis (RA)	Multiple groups	PTPRC rs10919563 A allele and FCGR2A homozygous and heterogynous genotypes are associated with poor response to anti-TNF therapy	(128)
Infliximab, Adalimumab, Etanercept	*NLRP3*	*NLRP3* g.247448734 G>C (rs10754558)	Rheumatoid Arthritis (RA)	European	Prevents the blockade of the interaction between TNF-α and the binding sites of the cell surface receptors for TNF-α	(129)
Infliximab	*TNF*	TNF g.31574705 C>T (rs1799724)	Crohn’s disease (CD)	Japanese	The T allele in rs1799724 showed a poor response	(130)
Infliximab	*TNF*	TNF -308G>A (rs1800629)	Inflammatory bowel disease (IBD)	European	The A allele in rs1800629 showed a poor response	(131)
Infliximab	*TNFRSF1A*	*TNFRSF1A g.*6341779 T>C (rs767455)	Crohn’s disease (IBD)	American	rs767455 was significantly associated with a lack of efficacy	(132)
TNF inhibitors*	*TNFRSF1B*	*TNFRSF1B g.*12207208 A>G (rs1061624), *TNFRSF1B* 12207235C>T (rs3397)	Rheumatoid arthritis (RA)	American	Patients with the TNFRSF1B rs3397C/C, TNFRSF1B rs1061622G/G, and TNFRSF1B rs1061631A/A genotypes showed increased risk of having a worse response to anti-TNF drugs	(133)
Etanercept	*TNF*	TNF -308G>A (rs1800629)	Rheumatoid arthritis (RA)	European	AA genotype is associated with decreased response to etanercept as compared to AG and GG	(134)
Adalimumab, Infliximab	*TNFRSF1B*	*TNFRSF1B g.*12207208 A>G (rs1061624)	Crohn’s disease (CD)	European	AA+AG genotype is associated with decreased response to Adalimumab and Infliximab as compared to GG	(135)
Infliximab	*TLR4*, *IL-6*, *IL18*, *TLR2*, *NLRP3*, *NFKB1A*, *JAK2*	*TLR4* g.117712004 G>A (rs5030728), *IL6* g.22720869 T>C (rs10499563), *TNFRSF1A* ‐609 G>T (rs4149570), *IL18* ‐137 G>C (rs187238), *TLR2* C>A (rs11938228), *NLRP3* C>T (rs4612666), *NFKBIA* 2758 G>A (rs696), *JAK2* T>C (rs12343867), *IL18* ‐607 C>A (rs1946518),	Inflammatory bowel disease (IBD)	European	Associated with beneficial response to TNF inhibitors in patients with IBD	(136)
Infliximab	*IL1B*	*IL1B* –3737G>AG>A (rs4848306), *IL6* –6331T>C (rs10499563)	Inflammatory bowel disease (IBD)	European	Both homozygous and heterozygous genotypes of these two SNPs are associated with a beneficial response to TNF inhibitors in UC patients	(123)
Adalimumab	*STAT4, PSORS1C1*, *PTPN2* and *TRAF3IP2*	*STAT4* g.191099907T>A (rs7574865), *PSORS1Cg.*31139584 C>A (rs2233945), *PTPN2* g. 12877061 A>C (rs7234029), *TRAF3IP2g.*111592059 C>A (rs33980500)	Rheumatoid arthritis (RA)	European	These SNPs are associated with an increase in response to Adalimumab	(137)
Infliximab, Certolizumab, adalimumab	*FAM114A2*	*FAM114A2g.*154063336 A>T (rs34767465)	Inflammatory bowel disease (IBD)	European	Presence of the rs34767465 variant is associated with non-response to TNF inhibitors	(138)
Etanercept, adalimumab, or infliximab	*TNFRSF1B*	*TNFRSF1Bg.*12192898 T>G (rs1061622)	Psoriasis	European	T allele in the rs1061662 variant is associated with a good response to etanercept, but not with adalimumab or infliximab	(139)
Infliximab	*IL1B*	*IL1B* g.112832813 G>A (rs1143634)	Inflammatory bowel disease (IDB)	European	C allele in rs1143634 is associated with poor response	(140)
Etanercept	*TNF*	TNF α -308G>A (rs1800629)	Crohn disease (CD), Inflammation, Psoriasis, Ankylosing spondylitis, Rheumatoid and Psoriatic Arthritis	European	Genotype AA decreased response where AG and GG increased response	(134)
Adalimumab	*MIF*	*MIF* g.23894205 G>C (rs755622)	Crohn’s Disease (CD)	European	Associated with better response to Adalimumab	(141)
Etanercept	*CYP2C9, CYP2D6, CYP3A5*	CYP2C9∗3, CYP2D6∗10, CYP3A5∗3	Ankylosing spondylitis	Chinese	Genotype CC of CYP2D6^∗^10 polymorphism and CYP3A5^∗^3 polymorphism is correlated with etanercept efficacy	(142)
TNF inhibitors*	*CTLA4, FBXL19, IL23R, SLC12A8, TAP1*	*IL23R* g. 67240275 AG/GG (rs11209026), *FBXL19* g. 30931304 GG (rs10782001), *CTLA4* g.203874196 AG (rs3087243), *SLC12A8* g.125092470 AA (rs651630), *TAP1* AG (rs1800453)	Psoriasis	European	Genetic polymorphisms in *IL23R, FBXL19, CTLA4, SLC12A8*, and *TAP1* are associated with paradoxical reactions in psoriasis patients treated with anti-TNFα drugs	(143)

TNF inhibitors*: Infliximab, Certolizumab pegol, Golimumab, Etanercept, and Adalimumab.

In RA, a study conducted on 1752 patients treated with Infliximab in the UK found that the *FTO* g.54026293G>A (rs7195994) was related to Infliximab response (116). Another study examined variants in *NKG2D* in 280 RA patients undergoing anti-TNF therapy. *NKG2D* g.10379727A>G(rs2255336) and *NKG2D* g.10372766C>G (rs1049174) variants showed significant associations of CC or GG genotypes to poor response (p=0.003, p=0.004 respectively). Moreover, the GG genotype was associated with non-response in etanercept-treated patients after 12 weeks (124). The genetic link to the response to TNFi has also been investigated in IBD patients. A study of an Italian cohort reported that IBD patients possessing a variant in *FCGR3A* exhibited diminished clinical response by the end of the induction period. Moreover, they observed a remarkable correlation between the *FCGR3A* variant and median Infliximab levels during maintenance therapy, revealing that patients with the wild-type genotype demonstrated elevated Infliximab levels compared to those with the variant allele. Additionally, individuals with the variant allele displayed an increased likelihood of developing antidrug antibodies (145). Another longitudinal study conducted on 132 Crohn’s disease patients identified that SNPs including *TLR2* g.153688371T>C(rs1816702) and *TLR2* c.597T>C (rs3804099) were linked with long-term response to Infliximab, whereas *IL6* c.6331T>C (rs10499563) correlated with supratherapeutic and infratherapeutic Infliximab levels (117). They also identified that genotypes AG and GG in *TNFRSF1B* has been associated with lower response to adalimumab and infliximab. In pediatric Crohn’s disease patients treated with Infliximab, studies reported that genotypes *TNFRSF1B* CC>CT (rs3397) was associated with the anti-Infliximab antibody production, suggesting its potential in identifying patients prone to immunogenicity (146). More variants were recently associated with long-term response to infliximab or adalimumab in IBD pediatric patients, including rs10508884 (*CXCL12*), rs2241880 (*ATG16L1*), and rs6100556 (*PHACTR3*); these three SNPs were linked to poor long-term response to TNFi (92). Certain other variants were associated with disease type or drug type; for instance, rs6908425 (*CDKAL1*) was associated with poor response in CD, while rs2188962 (*IRF1-AS1*), rs2241880 and rs6100556, were associated with worse outcomes in ulcerative colitis (UC). Moreover, rs2241880 was linked to poor response to both infliximab and adalimumab, whereas rs6100556 correlated with poor response to infliximab specifically (92). Furthermore, in a genetic association study cohort comprised of 474 IBD patients with European ancestry, two loci were found to be significantly associated with response to TNFi and were replicated with a *p <*1 x 10–^03^ in a validation cohort: rs116724455 in *TNFSF4/18* and rs2228416 in *PLIN2*. Allele C of rs116724455, located on chromosome 1 near the *TNFSF4* and *TNFSF18* genes, was strongly associated with nonresponse to TNFi (OR = 19.9, *p* = 4.79×10−⁸). Likewise, allele T of rs2228416 on chromosome 9 near the *PLIN2* and *HAUS6* genes, was also linked to nonresponse (OR = 5.25, *p* = 5.24×10−⁶) (147). Four other SNPs showed suggestive association with the response to TNFi; those include rs762787 (allele T) near *LTF*, *CCR5*, and *CCRL2*, rs9572250 (allele G) near *KLHL1*, rs144256942 (allele G) near *PROX1* and *RPS6KC1* and rs523781 (allele G) near *RORB* and *TRPM6* (147). In a very recent study, certain genetic variants were associated with the likelihood of achieving steroid-free remission (SFR) in a small cohort of European IBD patients (83 patients) treated with infliximab or adalimumab (148). Patients carrying the GG genotype of rs1800629 (*p* = 0.025) and the AA genotype of rs1061624 (*p* = 0.029) had significantly higher odds of achieving SFR compared to those who did not. In contrast, patients with the AA genotype of rs361525 or the CC genotype of rs767455 were significantly less likely to achieve SFR. Moreover, the allele A of rs1800629 was more frequent in patients who discontinued TNFi treatment and was linked to a higher risk of early therapy interruption (148).

Similar to RA and IBD patients, studies have identified genetic variants linked to variable response to TNFi in psoriasis patients; one example is the *TNFRSF1B* c.196T>G (rs1061622) SNP, which was associated with a higher risk of poor response to TNFi in psoriasis patients of European ancestry (139). Remarkably, the G allele of rs1061622 was found more frequent in patients carrying the allele HLA-CW6*0602 (149). In a more recent study, a candidate variant, rs1991820, was identified via a retrospective study approach involving 1849 psoriasis patients (149). Although it did not reach genome-wide significance, rs1991820 (an intronic variant of *KLK7* gene) showed a promising association with positive response to TNFi (*p* = 1.30 × 10^–6^). The study found that rs1991820 is significantly correlated with elevated expression of *KLK7* in the skin tissue of psoriatic patients and that TNFi treatment gradually reduced *KLK7* expression levels (149). In a study involving 312 ankylosing spondylitis patients treated with Etanercept, patients with specific genotypes (CYP2C9*3, CYP2D6*10, and CYP3A5*3) showed lower joint swelling, erythrocyte sedimentation rate, and C-reactive protein levels after 24 weeks of Etanercept treatment. The CYP2D6*10/*10 and CYP3A5*3/*3 diplotypes were associated with higher efficacy scores, suggesting a potential correlation between genetic variations and Etanercept treatment response in these patients (142). In one study of 74 Italian Behçet syndrome patients receiving anti-TNFα therapy, the *TNF* -308G>A (rs1800629) variant was found to be associated with treatment response; GG genotype prevalence was significantly higher among responders (86.2%) compared to non-responders (56.3%) (150). Well-characterized cohorts with larger samples size are needed for further validation of *TNF* -308G>A (rs1800629) as a predictive biomarker for TNFi response. A study on an Italian cohort found a significant association between the *TNF* -308G>A (rs1800629) and clinical remission without steroids in pediatric patients receiving infliximab therapy. Additionally, the study identified a potential link between carriers of the HLA-DQA1*05 and a higher risk of developing anti-TNFα immunogenicity (151). A previous study conducted on Psoriasis patients from the European population reported that variants in the *TNF* -857C and *TNFRSF1B* 676T genes were associated with positive response when treated with Etanercept. Moreover, no significant associations were found between variants in these genes and treatment outcomes for infliximab or adalimumab (121). Another study conducted on European population reported that variants in *IL1B* (rs1143623, rs1143627), *LY96* (rs11465996), *TLR2* (rs11938228, rs4696480) and *TLR9* (rs352139) were associated with response to Adalimumab, Infliximab and Etanercept (122). Furthermore, a recent study conducted on 738 European patients with IBD using a candidate gene approach identified 19 functional polymorphisms in genes involved in NFκB activation via TLR pathways, TNF-α signaling, and NFκB-regulated cytokines as significant predictors of response to anti-TNF therapy. The study highlighted that a genetically strong TNF-mediated inflammatory response was associated with favorable treatment outcomes, while also suggesting alternative cytokine targets such as IL-1β, IL-6, and IFN-γ for non-responders (136). These findings suggest that pharmacogenetic monitoring could become an essential tool in tailoring anti-TNF therapies for both pediatric and adult populations.

## 8 Clinical implications

Autoimmune disease treatment poses significant clinical and economic challenges, including decisions on when to start targeted therapy and which drug class to select. The decision of prescribing TNFi therapies is often driven by general guidelines (described below), pricing incentives, and habitual prescribing patterns (152). One study reported that 98.8% of participating rheumatologists (n= 248) expressed interest in a predictive test for inadequate TNFi response, noting that such a tool would influence their treatment decisions and patient management. The study also highlighted that 71% of rheumatologists were concerned that inadequate response to TNFi therapies result in patients paying for ineffective treatments. The current prescribing practices entail patients switching between multiple TNFi drugs before switching to drugs with different mechanisms of action (152, 153). This approach raises healthcare costs, extends high disease activity, and negatively impacts quality of life. According to the guidelines of the American College of Rheumatology, the physician’s decision to initiate or switch biologic DMARDs should consider a patient’s disease activity, prior DMARD use, and comorbidities (154). Studies investigating the predictors of biologic treatment in RA patients have shown variable findings (130). Some findings suggest that biologic initiation is influenced by previous glucocorticoid or non-biologic DMARD use (155). Other studies highlight the association between sociodemographic factors, such as lower income and older age, and accessibility to biologics. The differences in study design, population, and disease stage have led to inconsistent conclusions. While most research efforts in this area focused on initiating biologics, evidence on switching between biologics is scarce. When the optimal treatment is delayed, patients are subjected to a poor quality of life and several risks, including adverse events, disease progression, and chronic pain. The clinical applications of pharmacogenomics of TNFi entail personalized drug prescription and dosage based on the genetic profile of the patient, prediction of response prior to treatment initiation, and prediction of the risk of adverse events and immunogenicity before they occur, aiming to achieve personalized treatment and better health outcomes.

### 8.1 Individualized dosing

The relationship between the dosage and the serum concentration of biologics is not linear due to many implicated factors pertaining to the patient and the drug. Although a personalized approach based on genetic variation has not yet been developed for TNFi dosage, the association between genetic variation and drug dosage is well established. Genetic variations can affect the metabolism and clearance of drugs. By analyzing specific genes involved in drug metabolism, healthcare providers can tailor the dosage of TNFi to match the patient’s genetic profile. One of the promising examples of how personalized TNFi dosage can improve clinical outcomes is the Bayesian Dashboard approach, which is designed to consider pharmacokinetic factors that affect drug clearance (156). The model aims to provide individualized dosage plans tailored to maintain the treatment goal of drug concentration or trough levels as set by the physician. Due to the complex and multifactorial nature of the association between patient’s traits and the pharmacokinetics of TNFi, the Bayesian Dashboard approach models adjusting for these multi-factors. It achieves that by employing multiple functions to analyze new patient data and generating two predictions (157). Briefly, the model is first built using the pharmacokinetic parameters of the population, then it is refined by adding covariates data, such as the patient’s sex, BMI, disease activity, CRP level, albumin level, etc. A standard prediction is derived from the population parameters and patient traits. Afterwards, the model is updated with individual data to balance the population data and train the model on personalized data; the individual data includes information on drug concentration and the development of anti-drug antibodies (ADAs) during the course of the treatment. Then, the models can be averaged, or one model is selected to predict the dose and clearance of the drug in a personalized manner (individual prediction). For robust prediction, ADAs are included as a factor in the model because they can alter the pharmacokinetics of TNFi by binding to them and neutralizing them, or by increasing the drug clearance after forming a complex with TNFi (55). The standard prediction is beneficial for patients without prior drug concentration measurements, while the individual prediction aids in managing patients with existing drug concentration data (157). In regard to TNFi, several studies demonstrated the success of Bayesian Dashboard-based dosing in enhancing the clinical outcome of patients receiving Infliximab compared to standard dosing (158–160). For instance, 88% of patients with IBD maintained remission after precision dosing of Infliximab using a Bayesian-dashboard model compared to IBD patients following a standard therapeutic plan (64%) (160). In a real-life care situation involving 108 patients from a single center, a Bayesian Dashboard model was used to forecast personalized dose of infliximab. Based on the model, a higher dose was prescribed in 34 patients, a lower dose was prescribed in 9 patients, while infliximab administration was discontinued in 16 patients. As a result, the overall remission rate increased from 65.7% to 80.0% (158). Although the Bayesian-dashboard model does not involve pharmacogenomic variants, it serves as proof of the clinical utility and application of precision dosage on the outcomes of the disease.

The Bayesian Dashboard model has its advantages and disadvantages. As explained previously, the model includes multi-factors to accommodate the complexity of the link between these factors and drug dose and concentrations. Moreover, the model tends to be flexible, and this is reflected by the ability to refine it and update it with clinical values once available. Also, if a certain value was missing, the model can use the most recent value instead to perform the prediction. In other words, if CRP and albumin levels were not available, for example, they can be added at a later stage; and if CRP value was missing for a certain dose, the model will use the most recent one instead to predict the drug concentration during that period (157). Nevertheless, the Bayesian Dashboard model has some limitations. The model requires the values of 3 concentrations at different time points for the prediction to be maximally accurate. Furthermore, the robustness of the model is dependent on the size of the population data incorporated, and it was tested only with infliximab among the five TNFi. The cost-effectiveness of the model is also not assessed yet (157).

The clinical significance of pharmacogenomic variants on response to some drugs is solid and well-documented; however, the clinical significance of genetics variants associated with response to TNFi lacks validation before being implemented in clinical practice. Nonetheless, the genetic association to response to TNFi is extensively studied and reported. One example is the association reported by Salvadore-Martin et al, whereby SNP rs1816702 at *TLR2*g. 153688371(rs1816702) was identified as a potential biomarker for TNFi treatment plan in IBD pediatric patients (161). Four other SNPs, were also detected in association with subtherapeutic, supratherapeutic or absolute trough values of Adalimumab or Infliximab; the SNPs were *TLR4g.*117712004 G > A (rs5030728), *LY96g.*73989727C>G (rs11465996), *TNFRSF1B* g.12207235 C>T (rs3397), and CD14 g.140633331A> G (rs2569190) (161). Patients carrying these variants in this study exhibited different responses to the same TNFi; for instance, patients carrying allele T at *TLR2*g. 153688371(rs1816702) or allele G at CD14 g.140633331(rs2569190) had higher levels of serum adalimumab, while patients carrying allele C at *TNFRSF1B* g.12207235 C>T (rs3397) had lower serum levels of adalimumab. These associations became insignificant after removing patients with intensified drug dosage from the cohort; the authors referred this loss of association to low sample size (n=48), particularly because the impact of the first two SNPs was reported previously. The study also proposes that patients carrying genotype TT in *TLR2* g. 153688371(rs1816702) could benefit from adalimumab treatment more than infliximab (161). Another example is the association between variants in *NOD2* and response to TNFi; several studies evaluated this correlation, for example, Juanola et al, examined three SNPs in *NOD2* gene, whereby patients carrying any of the three variants needed an intensified TNFi regime due to loss of response (162). While such studies are promising, the clinical significance and utility of genetic polymorphisms in terms of response to TNFi still lack validation in large sample sizes to prove their robustness.

### 8.2 Identification of responders and non-responders

Pharmacogenomic insights can help identify patients who are likely to respond well to a particular drug, allowing for more targeted and effective treatment strategies. In parallel, early identification of non-responders can prevent unnecessary exposure to potentially ineffective medications. A plethora of genetic variants have been linked to the response to TNFi, and the variability of TNFi efficacy among patients was associated with specific genotypic variation. The association between HLA-B27 genotype and the response to TNFi has been examined in that regards for the treatment of axial spondyloarthritis, whereby HLA-B27 positivity predicted the reduction of Bath Ankylosing Spondylitis Disease Activity Index (BASDAI) within 6 months [OR = 2.11, 95% CI: 1.25, 3.56) (*P* = 0.005)]. In addition, HLA-B27 positivity predicted improved drug survival (HR = 1.76, 95% CI: 1.07, 2.89) (*P* = 0.027) (163). Nonetheless, HLA-B27 genotype’s utility and impact on predicting response is not straightforward; it was reported to have less weight in the prediction power compared to other factors such as the sex, inflammatory status and severity of the disease (112). However, HLA-B27 can aid in predicting the success of TNFi therapy after taking into consideration other implicated factors such as the gender and CRP levels of the patients (112). The mechanism of how HLA-B27 genotype is linked to TNFi response is not elucidated, but previous studies reported that patients with positive HLA-B27 had lower TNFα production by the T cells, and the mechanism behind it is unknown (164).

The known genetic associations to TNFi response have not been utilized in clinical settings yet; however, promising findings on the clinical utility of genotype have emerged. One illustration is generating Genetic Risk Scores (GRSs) based on a combination of clinical and genetic factors to predict response to TNFi; Barber et al. demonstrated that a GRS composed of 31 distinct SNPs can be used to predict primary non-response and durable response with higher accuracy than prediction based on clinical factors solely (90). Similarly, a risk model and a preliminary “anti-TNF refractory score” designed by Wang et al. that included clinical and genetic predictors, highlighted that adding the genetic factors enhanced the predictability of the model. The genetic compartment of the model involved 18 novel genome-wide significant loci in the *TNFSF4* gene, along with several other suggestive loci that correlated to the response to TNFi. The predictability of the model, represented by the area under the curve, raised from 0.72 to 0.89 upon adding the genetic markers to the model. The anti-TNF refractory score, which involved clinical factors along with the genotypes of the most significantly associated loci, differentiated between responders and non-responders to TNFi, with better accuracy metrics than when clinical data is used solely. After adding the genetic data, the specificity increased from 75% to 95%, the positive predictive value increased from 19% to 50%, the negative predictive value increased from 95% to 96%, while sensitivity exceptionally dropped from 61% to 56%. Hence, the study proposes the possible utility of such models as a precision medicine tool for predicting response to anti-TNF treatment, after validating the power of the model in an independent cohort (147). Furthermore, another research team proposed a model based on Gaussian Process Regression (GPR) (165). The model incorporates clinical, demographic, and genetic data of rheumatoid arthritis (RA) patients to anticipate variations in Disease Activity Score in 28 joints (DAS28). Due to heterogeneity among patients, the model matches the data of patients from the testing group to the data of patients in the training set to predict changes in DAS28 and classify patients as responders and non-responders. The model succeeded in predicting the response to TNFi of 78% of the patients (with an AUC of ~0.66). The GPR model works properly with heterogeneous datasets, commonly encountered in cross-sectional studies, in which disease variability and limited sample sizes are often the challenges. GPR addresses this by matching patients with similar conditions through its kernel function and predicts the response to treatment per subpopulations. Unlike complex machine learning algorithms that lack interpretability for clinical applications, GPR uses intuitive similarity-based modeling, and its results can be easily interpreted by the physicians. Moreover, the model’s additive design facilitates integrating new features with minimal parameter adjustment and provides confidence intervals, which are valuable for physicians. However, GPR lacks built-in feature selection available in linear methods. To overcome this disadvantage, the study used a preselected set of genetic features validated through cross-validation with clinical data. Although the clinical features had a higher prediction power than the genetic features, the genetic data undeniably enhanced the prediction power of the model when it was combined with the clinical data (165). One drawback of the GPR model is lack of generalization, as it was designed using data of European patients, so the model needs to be refined before it is used for other populations (165).

Apart from genetic markers, the clinical utility of transcriptomics data was also evaluated, in which a machine learning prediction model for response to TNFi was designed based on gene expression data (166). The study tested various machine learning models (linear, non-linear, and kernel-based) to predict response to TNFi based on clinical data, flow cytometry data, proteomic data, and transcriptomic data. The models showed high prediction power; particularly, the transcriptomics-based model had a higher power in classifying non-responders than the clinical-based and proteomics-based models (166). The findings of these models should be validated in a larger cohort as the sample size in this study was small, especially the non-responder cohort; also, only female patients were included in this study to reduce heterogeneity, thus the proposed models need to be tested using male samples to examine its generalizability (166). The strength of genetic markers over clinical features is also highlighted in another study in which clinical data and imputed gene expression profiles from genome-wide genotype data were utilized to predict non-durable (short-term) response to TNFi through machine learning models. The top three significant features were the genetic markers (*DPY19L3, GSTT1*, and *NUCB1*) and not the clinical features (167).

Moreover, the power of combining genetic and transcriptomic data was also assessed in terms of predicting response to TNFi, whereby gene expression and SNPs data derived from RNA sequencing were combined along with clinical data to design a multifaceted algorithm comprised of 70 features in total (168). Although SNPs information is typically derived from whole genome sequencing (WGS), RNAseq data can also offer valuable insights into functionally active SNPs. In other words, SNP identification using RNA-seq data of over-expressed genes offers advantages over WGS, as it focuses on transcribed regions, which enhances detecting functionally significant SNPs located in coding exons, untranslated regions (UTRs), and introns (169). Upon combining these SNPs with gene expression profiles, they have the potential to significantly improve the accuracy and effectiveness of molecular signature predictions. This study involved SNPs derived from RNA-seq data and that are linked to rheumatoid arthritis and are associated with differential gene expression in peripheral blood mononuclear cells. Out of the 70 features used to train the prediction model, it used 23 features to classify responders and non-responders, including 8 transcripts, 10 SNPs, CRP and anti-CCP levels, gender, BMI, and patient disease assessment. The study involved 58 American female RA patients in the discovery cohort, while the training and validation cohorts were comprised of 145 and 175 patients, respectively. The model differentiated patients who are likely to have a poor response to TNFi with a positive predictive value (PPV) of 89.7% (95% CI 79.0–95.7%), specificity of 86.8% (95% CI 72.4–94.1%), and sensitivity of 50.0% (95% CI 40.8–58.7%). The authors in this study have not addressed the low sensitivity of the model although it misses half of the true non-responders. The study highlights that 70% of the unstratified patients didn’t achieve a 50% improvement after receiving TNFi treatment and that the patients didn’t achieve a 50% improvement after receiving TNFi treatment, therefore, the model could have saved half of them from taking TNFi by predicting the inefficacy of the treatment in these patients (168). Despite the model’s strong PPV and specificity, its low sensitivity highlights the need to refine the model to better identify all true non-responders; increasing sensitivity could be achieved by integrating additional biomarkers or expanding the dataset to include larger cohort. Several other research groups attempted to design prediction tools/models to anticipate the response to anti-TNFα treatment in the realm of precision medicine; however, those models still need further testing, validation in large cohorts, and enhancements before they can generate optimal prediction and be used in clinics.

Multi-omic analyses have also provided key insights into treatment responses in psoriasis. For example, a previous study applied a multi-omics approach to characterize treatment response to the Etanercept in patients with severe psoriasis (170). By integrating transcriptomic, proteomic and systems biology analyses across blood and skin at multiple timepoints, they revealed molecular signatures linked to therapeutic response. Importantly, the expression of TNF-regulated genes in both lesional skin and peripheral blood showed significant correlation with clinical outcomes. Despite conventional skepticism toward blood-based biomarkers in dermatology, the study highlighted the predictive potential of baseline blood samples. These findings provide not only valuable candidate biomarkers but also designed a scalable analytical framework for larger biomarker discovery efforts such as the full-scale Psoriasis Stratification to Optimise Relevant Therapy (PSORT) study (170).

### 8.3 Predicting and preventing adverse events and/or immunogenicity

As highlighted **Table 1**, genetic variants, such as variants in the HLA region, have been identified to be associated with adverse reactions after TNFi therapy. Immunogenicity is defined as an immune response triggered by a biologic drug that manifests as production of anti-drug antibodies (ADAs). It is one of the mechanisms behind treatment failure, by which ADAs would either neutralize the binding site of the biologic drug or increase its clearance, leading to lower therapeutic levels of the drug. As discussed earlier, previous evidence shows that immunogenicity has a genetic component, and several genetic variants, such as HLA variants, were associated with the development of ADAs in response to TNFi. Variants in *CXCL12*, *IL-10*, and *Fc gamma receptor (FCGR3A)* genes were also linked to ADAs in patients receiving TNFi. Therefore, predicting immunogenicity in susceptible patients can assist physicians in providing a personalized treatment plan to minimize the risk. A recent study showed that Crohn’s disease patients who had free antibodies to Infliximab responded poorly to an intensified drug dose compared to patients without free antibodies to the drug. The findings proposed that free antibodies level, but not total antibodies, could be a plausible biomarker of response to Infliximab. Furthermore, ADAs developed in response to Infliximab or Adalimumab were previously used to predict the discontinuation of treatment within 24 months after initiating the therapy with sensitivity of 62% and 79%, and specificities and positive predictive values of 100% (171).

However, immunogenicity does not always lead to drug inefficacy, which makes the prediction of drug inefficacy based on ADAs titer challenging. Nevertheless, ADAs have been linked to several adverse reactions. For example, a higher incidence of reactions to infusion and in the injection sites were reported in patients who developed ADAs compared to their counterparts who didn’t have ADAs (172). Moreover, switching to another biologic drug has been found effective in some patients who developed ADAs to specific biologics, especially because biologics have different immunogenicity levels. The immunogenicity levels of different TNFi vary, with some agents showing higher rates of antibody formation compared to others (54). These differences can impact treatment efficacy and the risk of adverse reactions in patients with inflammatory diseases such as rheumatoid arthritis, psoriasis, and inflammatory bowel disease. For example, studies have shown that monoclonal antibody-based TNFi, such as Infliximab and Adalimumab, tend to have higher immunogenicity rates compared to soluble receptor-based TNFi like Etanercept (173). In addition to genetic variants, transcriptomic data, such as mucosal TNFα expression level, was also reported to be used as a possible biomarker of response to TNFi and generation of ADAs. The subdued expression of mucosal TNFα at diagnosis was correlated with better response to TNFi (174). Although all of this sounds promising, to our knowledge, no model has been designed yet to examine the utility of genetic variants in predicting immunogenicity or adverse events in response to TNFi, and the associated genetic variants are still lacking validation before genetic testing is implemented in the clinics for this purpose.

### 8.4 Key challenges that impede validation of biomarkers

Despite the compelling evidence of the impact of genetic variants on response to TNFi, genetic testing has not been integrated into the clinical practice of prescribing TNFi due to a lack of replication and validation of the identified genetic biomarkers. The challenges behind this could be several. A quite obvious reason is the heterogeneity among patients in terms of medical conditions, lifestyle, and treatment plans. The treatment plan varies based on the condition of each patient, and most patients end up taking concomitant drugs; this type of inter-individual variability limits the power of the study. Other study design-related challenges that face the discovery of robust genetic biomarkers are the sample size and lack of standardization. For instance, to our knowledge, none of the GWAS and meta-analyses that have been published on the response of TNFi in rheumatoid arthritis exceeded 3000 participants; the largest cohort that a meta-analysis assessed was 2706 patients (175). This limitation likely contributes to the failure to replicate earlier candidate gene findings. Furthermore, unstandardized handling procedures could also affect the validity and replication of identified biomarkers, such procedures include collection, transportation, processing, and storage of samples. Another factor could be the limitation of clinical biomarkers in reflecting the true response to TNFi; for example, the disease score DAS-28, which is used to assess the disease activity in rheumatoid arthritis, has been criticized with some limitations despite its practicality and validity in the practice of rheumatology clinics (176, 177). The limitation primarily arises from the “general health” component incorporated in DAS-28 calculations; the general health is a subjective assessment provided by the patient, which makes it more prone to bias and measurement errors. Studies have reported cases where the general health score was high while all other components of DAS-28 didn’t reflect an active disease (178). While it is important to account for patient-reported assessment of the disease severity, given that autoimmune diseases are multifactorial, other more reliable patient-reported measures should be explored to replace the general health score in DAS-28 assessment. Furthermore, discrepancies in swollen-joint count have been reported due to differences in the training and experience of the physician assessing it, a lack of standardized assessment techniques, obscure definitions of swelling, or variations in the severity of joint deformities (177). Moreover, an overlooked factor could be the lack of patients’ adherence to TNFi; a previous study found that around 27% of patients receiving TNFi reported that they did not follow their treatment regimen, ending up with failing to respond (179). While reasons of non-adherence were not known in that study, another study discussed that non-adherence to TNFi could be for practical reasons or for perceptions that patients have regarding the treatment; the practical reasons include the administration route of the treatment and the cost of the treatment; whereas, the perceptual reasons include worries of side effects and unawareness of the patient about the importance of adherence and the consequences of non-adherence (180).

## 9 Future directions

As discussed in the previous section, several limitations need to be addressed before achieving a valid personalized approach for patients on TNFi. Concisely, the main factors to be addressed are reliability, standardization, and sufficient statistical power to discern authentic and robust associations. Investigating a sole aspect is not enough to explain the variability in response to TNFi. Therefore, future study designs must develop models that integrate genetic, clinical, demographic, and environmental data to capture the intricate heterogeneity among patients and achieve robust accuracy, specificity, and sensitivity values. This comes hand-in-hand with increasing sample size and studying diverse populations. As a matter of fact, in 2020 the pharmacogenomics GWAS represented around 7% only of all published GWAS, and the majority of these studies predominantly focused on European populations (181). The GWAS on response to TNFi is even more scarce; hence, large-scale GWAS of response to TNFi are needed in diverse and less represented populations. Moreover, patient stratification is another area that needs improvement, whereby previous studies primarily aimed to distinguish between responders and non-responders whilst grouping patients of various response, such as optimal response and moderate response, in one category under “good responders”. This might have been due to limited sample size, which reiterates the necessity of larger cohorts to achieve significant results. Similarly, it is equally essential to predict primary or secondary non-response to TNFi. As a matter of fact, secondary non-response results in many cases from immunogenicity-related causes, and models that predict immunogenicity are still lacking. Hence, patients who will develop a secondary failure of TNFi might be faultily categorized as responders. Designing models that can predict immunogenicity and secondary non-response would be helpful for the physicians in prescribing a less immunogenic TNFi or other complementary treatments that can prevent the probability of developing antidrug antibodies. In fact, the temporal change in response to TNFi necessitates longitudinal study designs to assess the response to treatment, particularly because a significant proportion of patients tend to lose their response to TNFi almost a year after the initiation of the treatment. Whilst most studies would evaluate patients’ response within 3 to 6 months, more studies focusing on the long-term response are required to capture the variability in response spectrum among patients and achieve a tailored treatment plan. Furthermore, from a precision medicine approach, future investigation of the mechanisms behind the failure of response to TNFi is also crucial, especially in the absence of cure for autoimmune diseases, whereby non-responders to TNFi suffer from a poor quality of life.

Emerging technologies such as machine learning (ML), single-cell genomics, and multi-omics, hold great promise in revolutionizing the prediction of TNFi response in patients. Previous studies have employed machine learning models to predict TNF inhibitor use in rheumatologic conditions (165–167). These studies typically analyze baseline clinical and laboratory data to identify patients likely to benefit from early TNF inhibitor therapy. ML approaches such as artificial neural networks (ANN) (165), have demonstrated superior predictive performance compared to traditional methods like logistic regression and support vector machines. ML with its ability to process large, complex datasets, can uncover non-obvious patterns within genetic, clinical, and environmental data (182). By training models on patient-specific characteristics, ML algorithms can predict who is more likely to respond to TNFi treatment, allowing for more personalized and effective therapeutic strategies (166).

Moreover, single-cell genomics enables the study of individual cells within heterogeneous tissue samples, offering detailed insights into immune cell dynamics at the molecular level. This technology is especially valuable in identifying rare or previously undetected cell populations that may play critical roles in drug response or resistance (183). By isolating and analyzing immune cells from patients on TNFi therapy, single-cell genomics can pinpoint specific immune pathways or cell types contributing to treatment efficacy or adverse effects, ultimately guiding better-targeted therapies. Furthermore, multi-omics approaches, which integrate data from genomics, transcriptomics, proteomics, and metabolomics (184), provide a comprehensive view of the biological processes that underlie drug response. These approaches can help identify novel biomarkers that predict treatment outcomes and uncover complex interactions between genes, proteins, and metabolites that influence how patients respond to TNFi. By combining data across multiple biological layers, multi-omics can reveal the full complexity of TNFi treatment responses, helping to tailor therapies to individual patients based on their unique molecular profiles. Together, these technologies represent the future of TNFi response prediction, offering the potential for more accurate, personalized treatment plans that improve patient outcomes while minimizing adverse effects. Moreover, the successful integration of TNFi response prediction into clinical practice requires collaboration between regulatory agencies, clinicians, and patients. Regulatory agencies like the FDA and EMA must validate predictive biomarkers and diagnostic tools, ensuring they meet safety and efficacy standards. Clinicians need to incorporate these tools into daily practice, interpreting complex data to make informed decisions tailored to individual patients. Patients must be educated on how genetic testing, and predictive models can help in tailoring a personalized treatment plan and adhering to it is part of the therapy; this is important to ensure informed consent and active participation in the decision-making process. Ultimately, close collaboration among these stakeholders will ensure the effective and safe implementation of personalized TNFi therapies.

## 10 Conclusion

Although TNFα inhibitors have improved the quality of life of many patients with autoimmune diseases, a significant proportion of patients remain in a progressive disease state and poor quality of life due to the failure of anti-TNFα therapy. Previous evidence demonstrated that the variability in response to TNFi is associated with genetic variants. Therefore, integrating a pharmacogenomic therapeutic approach seems to be promising for personalized treatment plans. As scientific research advances in this area, identifying robust genetic biomarkers to predict the response and the risk of adverse reactions to TNFi would ultimately improve the quality of care for patients with autoimmune diseases.
